# POINTER: study protocol for a phase 2b, randomised, placebo-controlled, double-blind, parallel group dose-finding clinical study to evaluate the efficacy of RMC-035 on renal function and safety, in participants at high risk for kidney injury, following open-chest cardiac surgery

**DOI:** 10.1186/s13063-025-09124-x

**Published:** 2025-10-28

**Authors:** Alexander Zarbock, Christian Strauss, Maxime Laflamme, Andrej Myjavec, Johannes Böhm, Jan Burkert, C. David Mazer, Benoit de Varennes, Antonino Ginel Iglesias, Klaus Matschke, Tobias E. Larsson, Michael Reusch, POINTER study group

**Affiliations:** 1 Department of Anaesthesiology, Intensive Care and Pain Medicine, University Hospital Münster, Münster, Germany; 2 Institut Universitaire de Cardiologie et de Pneumologie de Québec, Québec, Canada; 3 Department of Cardiac Surgery, Faculty of Medicine, Charles University, University Hospital in Hradec Kralove, Hradec Kralove, Czech Republic; 4 Department of Cardiovascular Surgery, Institute Insure, German Heart Center Munich, Technical University of Munich, Munich, Germany; 5 Fakultni Nemocnice V Motole, Prague, Czech Republic; 6 St. Michael’s Hospital, University of Toronto, Toronto, Canada; 7 McGill University Health Centre - Royal Victoria Hospital, Montréal, Canada; 8 Hospital de La Santa Creu I Sant Pau, Barcelona, Spain; 9 Klinik für Herzchirurgie, Herzzentrum Dresden Universitätsklinik, Dresden, Germany; 10 Guard Therapeutics International AB, Stockholm, Sweden

**Keywords:** Acute kidney injury, AKI, Cardiopulmonary bypass, Ischemia–reperfusion injury, IRI, Alpha-1-microglobulin, A1M, Major adverse kidney events, MAKE

## Abstract

**Background:**

Cardiac surgery with cardiopulmonary bypass invariably induces renal stress and risk of irreversible kidney function loss, with no approved drug treatments. RMC-035, a recombinant human alpha-1-microglobulin with potent heme-binding and antioxidant capacity, has shown promising long-term kidney-protective effects in a phase 2a trial of patients undergoing cardiac surgery. The primary objective of this phase 2b dose-optimisation trial is to demonstrate that RMC-035 (pooled dose groups) is superior to placebo in preserving renal function at 90 days after surgery.

**Methods:**

This randomised, blinded, placebo-controlled, multicentre study evaluates the efficacy and safety of RMC-035 among approximately 161 high-risk patients undergoing cardiac surgery who are randomised into one of three treatment groups in a 2:2:3 ratio: RMC-035 (30 mg or 60 mg) or placebo. The study drug is administered via three intravenous infusions, with the first dose given intraoperatively, followed by additional doses at 6 and 24 h, respectively. The primary endpoint is the change in estimated glomerular filtration rate (eGFR) from baseline (pre-surgery) to Day 90. Important secondary endpoints include the incidence of major adverse kidney events at Day 90 and short-term outcomes reflecting changes in renal filtration markers up to Day 7. Safety assessments encompass adverse events, vital signs, electrocardiograms and routine safety laboratory tests. Additional evaluations include pharmacokinetics, anti-drug antibodies and immunological biomarkers.

**Discussion:**

This multicentre, multinational phase 2b trial, will assess the change in eGFR within 90 days of the first dose, providing additional evidence of the long-term kidney-protective potential of RMC-035 in patients undergoing cardiac surgery at high risk for kidney injury. Trial outcomes will inform the preferred dose, dosing regimen, and benefit-risk profile related to cardiac surgery for a future pivotal phase 3 trial.

**Trial registration:**

The trial was registered June 20, 2024, at Clinicaltrials.gov (NCT06475274 https://clinicaltrials.gov/study/NCT06475274). The trial including the participating EU countries is also registered with the EUCT number 2024–510658-28 under https://euclinicaltrials.eu/search-for-clinical-trials/?lang=en&EUCT=2024-510658-28-00.

The first patient was enrolled August 26, 2024.

**Supplementary Information:**

The online version contains supplementary material available at 10.1186/s13063-025-09124-x.

## Administrative information

**Table:** 

Title {1}	A phase 2b, randomised, placebo-controlled, double-blind, parallel group dose-finding clinical study to evaluate the efficacy of RMC-035 on renal function and safety, in participants at high risk for kidney injury following open-chest cardiac surgery (POINTER)
Trial registration {2a and 2b}	NCT06475274, clinicaltrials.gov; 2024–510658-28, euclinicaltrials.eu
Protocol version {3}	02 July 2024; version 2.1
Funding {4}	The POINTER trial is funded by Guard Therapeutics International AB, Stockholm, Sweden
Author details {5a}	^1^Alexander Zarbock, Department of Anaesthesiology, Intensive Care and Pain Medicine, University Hospital Münster, Münster, Germany (Coordinating Investigator) ^1^Christian Strauss, Department of Anaesthesiology, Intensive Care and Pain Medicine, University Hospital Münster, Münster, Germany ^2^Maxime Laflamme, Institut Universitaire de Cardiologie et de Pneumologie de Québec, Québec, Canada ^3^Andrej Myjavec, Department of Cardiac Surgery, Charles University, Faculty of Medicine and University Hospital in Hradec Kralove, Hradec Kralove, Czech Republic ^4^Johannes Böhm, Department of Cardiovascular Surgery, Institute Insure, German Heart Center Munich, Technical University of Munich, Munich, Germany ^5^Jan Burkert, Fakultni Nemocnice v Motole, Prague, Czech Republic ^6^C. David Mazer, St. Michael’s Hospital, University of Toronto, Toronto, Canada ^7^Benoit de Varennes, McGill University Health Centre—Royal Victoria Hospital, Montréal, Canada ^8^Antonino Ginel Iglesias, Hospital de la Santa Creu i Sant Pau, Barcelona, Spain ^9^Klaus Matschke, Klinik für Herzchirurgie, Herzzentrum Dresden Universitätsklinik, Dresden, Germany ^10^Tobias E. Larsson, Guard Therapeutics International AB, Stockholm, Sweden ^10^Michael Reusch, Guard Therapeutics International AB, Stockholm, Sweden
Name and contact information for the trial sponsor {5b}	Sponsor: Guard Therapeutics International AB Nybrogatan 34 114 39 Stockholm Sweden medical@guardtherapeutics.com
Role of sponsor {5c}	The sponsor is responsible for funding of the trial and for trial design, collection, management, analysis, and interpretation of data, and writing of the report. The sponsor will also supply study intervention. The sponsor will comply with the requirements for publication of trial results. In accordance with standard editorial and ethical practice, the sponsor will generally support publication of multicentre studies only in their entirety and not as individual site data, in collaboration with the coordinating investigator

## Introduction

### Background and rationale {6a}

Cardiac surgery involving cardiopulmonary bypass (CPB) is associated with renal stress, frequently resulting in tissue damage. Such injury can lead to a clinically significant, serious, and irreversible decline in renal function, with a reported incidence of up to 30% in high-risk populations [1].

Determining the presence and extent of potential tissue injury based solely on short-term variations in serum creatinine (SCr) or an acute kidney injury (AKI) diagnosis per clinical guidelines is however challenging, often leading to an underestimation of the actual injury [2]. Furthermore, renal injury may be obscured by post-operative improvements in cardiac output and renal blood flow, along with activation of the functional renal reserve, which could mask nephron loss through hyperfiltration within remaining nephrons [3].

The pathophysiology of kidney stress and injury associated with cardiac surgery is complex and multifactorial, encompassing factors such as renal ischemia, heme toxicity, inflammatory responses, oxidative stress, and cytokine release, among others. In this regard, ischemia–reperfusion injury and heme toxicity are recognized as key contributors to cardiac surgery-associated kidney injury [4].

The high prevalence of clinically meaningful kidney function loss after cardiac surgery, with no approved drug treatments, underscores a significant unmet medical need. This is especially critical for patients with chronic kidney disease (CKD), who are prone to progressive kidney function decline and at increased risk of end-stage renal disease (ESRD). Novel therapeutic strategies to prevent or mitigate irreversible kidney function loss in this setting are therefore warranted [5].

Importantly, the endogenous protein alpha-1-microglobulin (A1M) possesses potent antioxidative and heme-binding properties, protecting cells and tissues from oxidative stress-induced damage, including injury from free radicals, reactive oxygen species and free heme [6]. To unlock its therapeutic potential, a modified human A1M protein (RMC-035) was engineered, demonstrating improved physicochemical properties over its native counterpart. Preclinical studies have shown RMC-035 to be consistently effective across a range of disease models, including those relevant to cardiac surgery [7].

RMC-035 has also been evaluated in clinical trials, including the phase 2 AKITA trial, which enrolled cardiac surgery patients at increased risk for AKI. Notably, RMC-035 improved kidney-related secondary outcomes compared to placebo, including estimated glomerular filtration rate (eGFR) and major adverse kidney events at Day 90 (MAKE_90_). Although the primary outcome – AKI within 72 h after surgery – was not met, the analysis was confounded by a short-term, reversible increase in SCr at the highest RMC-035 dose/exposure levels. This finding aligns with toxicological evaluations demonstrating kidney tubular protein overload when exceeding the maximum tubular capacity for RMC-035 uptake [8].

To optimize the benefit-risk profile of RMC-035 before a pivotal Phase 3 trial, a Phase 2b dose-optimization trial is underway, with key design elements presented herein. This clinical trial [NCT06475274; EU CT number 2024–510658-28] aims to evaluate the efficacy of RMC-035 at two different dosage strengths, focusing on its impact on long-term kidney function in cardiac surgery patients at increased risk for AKI.

### Objectives {7}

The purpose of this trial is to determine the optimal dosing regimen of RMC-035 for preserving long-term renal function, as assessed by changes in eGFR by Day 90, in patients undergoing cardiac surgery at high risk for kidney injury.

The primary objective is to evaluate the efficacy of RMC-035 (pooled doses of 30 mg and 60 mg) compared to placebo for preserving post-operative renal function at Day 90.

Key secondary objectives include assessing the efficacy of RMC-035 on eGFR and MAKE_90_, evaluated both by pooling RMC-035 dose arms and for each individual dose level. MAKE is a composite endpoint consisting of death, any new renal replacement therapy (RRT) after surgery, or sustained loss of kidney function defined as 25% or greater decline in eGFR from baseline.

Other secondary objectives involve evaluating the effects of RMC-035 on renal function at Days 7 and 60, as well as on MAKE at Day 60 (MAKE_60_). The trial will also investigate the efficacy of RMC-035 on postoperative changes in renal filtration markers, specifically SCr and cystatin C (CysC), up to Day 7. Furthermore, the development and characteristics of anti-drug antibodies (ADA) will be assessed for both pooled doses of 30 mg and 60 mg, as well as for each individual dose level of RMC-035.

Exploratory objectives will focus on changes in immunologic biomarkers and the pharmacokinetics of RMC-035.

The safety profile of RMC-035 will be evaluated across pooled doses as well as individual dose levels. The endpoints corresponding to these objectives are detailed in the Outcomes section.

### Trial design {8}

Phase 2b, randomised, double-blind, parallel group, multi-centre clinical study that will evaluate two different dose levels of RMC-035 compared to placebo in a 2:2:3 ratio in approximately 161 participants undergoing open-chest cardiac surgery at high risk for kidney injury.

## Methods: participants, interventions and outcomes

### Study setting {9}

Patients presenting at 19 large cardiovascular surgery units in Germany, Spain, and the Czech Republic, as well as in Canada, will be enrolled. A list of trial collaborators can be found in [Table 1].

**Table 1:** Collaborators in the POINTER study group

Type of collaboration	First and middle name	Surname
Study site; Coordinating Investigator	Alexander	Zarbock
Study site	Belén	Adrio Nazar
Study site	Johannes	Böhm
Study site	Andreas	Böning
Study site	Craig	Brown
Study site	Jan	Burkert
Study site	Benoit	de Varennes
Study site	Antonino	Ginel Iglesias
Study site	Maxime	Laflamme
Study site	Klaus	Matschke
Study site	C David	Mazer
Study site	Ignacio	Munoz Carvajal
Study site	Andrej	Myjavec
Study site	Nicolas	Noiseux
Study site	Guillermo	Reyes Copa
Study site	Christian	Strauss
Study site	Gabor	Szabo
Study site	Matthias	Thielmann
Study site	Marc	Vives Santacana
Sponsor	Tobias E	Larsson
Sponsor	Michael	Reusch
Sponsor	Sara	Thuresson

### Eligibility criteria {10}

Potential trial patients are screened at cardiothoracic surgery units for eligibility criteria as per [Table 2] after providing written informed consent to the trial site’s principal investigator or sub-investigator. The study includes adult patients of both sexes who are scheduled for non-emergent coronary artery bypass graft (CABG) surgery, valve surgery (whether single or multiple valves), or combination of CABG and valve surgery, or ascending aorta aneurysm surgery that requires the use of cardiopulmonary bypass. To qualify for inclusion, patients must present with at least one predisposing risk factor for AKI based on the type of surgery planned; see [Table 2]. Key exclusion criteria encompass patients with severe renal impairment, defined as an eGFR of less than 30 mL/min/1.73m^2^, those scheduled for emergent surgeries, off-pump surgeries, or patients who have required mechanical circulatory support within one week prior to the surgery.

**Table 2:** Study eligibility criteria

**Inclusion criteria**
Participants are eligible to be included in the study only if all of the following criteria apply:
1. Age is ≥ 18 and < 85 years at the time of signing the informed consent
2. eGFR is ≥ 30 ml/min/1.73m^2^ (at screening) using the CKD-EPI 2021 equation with SCr [9]
3. Scheduled for non-emergent surgery of any or several of the following types with use of CPB:
a. CABG surgery
b. Valve surgery (single or multiple valves)
c. Ascending aorta aneurysm surgery
4. Risk factors for kidney injury are present (at screening) as specified below:
a. eGFR is < 60 mL/min/1.73m^2^ with or without additional risk factors from list (1) to (8) below present, regardless of whether single type or combined surgery is planned
b. If eGFR is ≥ 60 mL/min/1.73m^2^
i. A combined surgery is scheduled AND at least one risk factor for kidney injury from list (1) to (8) below is present
ii. One type of surgery is scheduled AND at least two risk factors for kidney injury from list (1) to (8) below are present
Risk factors for AKI:
(1) Documented history of LVEF < 35% at any time during the 3-month period before or at the time of screening as assessed by either echocardiography, cardiac MRI or nuclear scan
(2) Repeat surgery/history of previous open chest cavity cardiac surgery with or without CPB
(3) Confirmed diagnosis of T2DM at least 3 months prior to screening AND ongoing treatment with an approved anti-diabetic drug
(4) Age ≥ 70 years at the time of screening
(5) Documented history of NYHA class II or higher at any time during the
(6) 3-month period before or at the time of screening
(7) Documented history of AKI as per KDIGO criteria longer than 3 months before date of screening, independent of the etiology of AKI
(8) Documented history of anaemia with haemoglobin ≤ 11 g/dL at any time during the 3-month period before or at the time of screening
(9) Documented history of albuminuria, defined as UACR > 100 mg/g in a spot urine sample or > 100 mg/24 h in a 24-h urine collection at any time during the 3-month period before or at the time of screening
5. Female Participants:
• A female participant is eligible to participate if she is not pregnant or breastfeeding, and one of the following conditions applies:
a. Is a WONCBP
b. Is a WOCB) and using a contraceptive method that is highly effective (with a failure rate of < 1% per year), with low user dependency, during the study intervention period and for at least 7 days after the last dose of study intervention
• A female participant is eligible to participate if she agrees not to donate ova during the study intervention period and for at least 7 days after the last dose of study intervention
• A WOCBP must have a negative highly sensitive pregnancy test (serum) within 48 h before the first dose of study intervention
• The investigator is responsible for review of medical history, menstrual history, and recent sexual activity to decrease the risk for inclusion of a woman with an early undetected pregnancy
6. Male Participants:
• Male participants are eligible to participate if they agree to the following during the study intervention period and for at least 7 days after the last dose of study intervention:
a. Refrain from donating fresh unwashed semen
b. And either (i) or (ii) below:
i. Be abstinent from heterosexual intercourse as their preferred and usual lifestyle (abstinent on a long term and persistent basis) and agree to remain abstinent
ii. Must agree to use contraception/barrier as detailed below:
Agree to use a male condom and should also be advised of the benefit for a female partner to use a highly effective method of contraception as a condom may break or leak when having sexual intercourse with a woman of childbearing potential who is not currently pregnant
Agree to use a male condom when engaging in any activity that allows for passage of ejaculate to another person
7. Capable of giving signed informed consent which includes compliance with the requirements and restrictions listed in the informed consent form and in the study protocol
8. Participant agrees not to participate in another interventional study from the time of signing the informed consent until the end-of-study visit
**Exclusion criteria**
Participants are excluded from the study if any of the following criteria apply:
1. Any medical condition that in the opinion of the investigator makes the participant unsuitable for study participation. This includes participants unable to give their informed consent
2. Scheduled for emergent surgeries (eg, aortic dissection)
3. Scheduled for CABG and/or valve surgery and/or ascending aorta aneurysm surgery combined with additional non-emergent cardiac surgeries (eg, congenital heart defects). Scheduled additional left atrial appendage closure surgery or maze surgery is not an exclusion criterion
4. Scheduled to undergo TAVI or TAVR, or off-pump surgeries or LVAD implantation
5. Experiences a cardiogenic shock or hemodynamic instability which require inotropes or vasopressors or other mechanical devices such as IABP within 24 h prior to surgery
6. Requires any of the following within one week prior to surgery: defibrillator or permanent pacemaker, mechanical ventilation, IABP, LVAD, other forms of MCS
7. Diagnosed with AKI (as defined by KDIGO criteria [10]), within 3 months prior to surgery
8. Requires cardiopulmonary resuscitation within 14 days prior to cardiac surgery
9. Ongoing sepsis (as defined by SEPSIS-3 guidelines [11]), within the past 2 weeks or, in the opinion of the investigator, an untreated diagnosed clinically significant infection (viral or bacterial) prior to or at screening and before randomisation
10. ALT or AST ≥ 3.0 × ULN
11. Total bilirubin ≥ 2.0 x ULN (Participants with Gilbert’s syndrome can be included with total bilirubin ≥ 1.5 × ULN as long as direct bilirubin is < 1.5 × ULN)
12. History of solid organ transplantation
13. History of RRT
14. Severe allergic asthma defined as confirmed diagnosis of asthma poorly controlled while receiving high-dose inhaled corticosteroid treatment, or with requirement of a high level of treatment to maintain control
15. Chronic immunosuppressive treatment that may have an impact on kidney function as assessed by the medical monitor
16. Ongoing chemotherapy or radiation therapy for malignancy that may have an impact on kidney function as assessed by the medical monitor
17. Current enrolment or past participation within the last 90 days (or within 5 half-lives of an investigational study treatment, whichever is longer) before signing of consent in any other clinical study involving an investigational study treatment
18. Has previously received RMC-035
19. Hypersensitivity to any of the study interventions, components thereof including excipients, or drug or other allergy that, in the opinion of the investigator, contraindicates participation in the study

*AKI* acute kidney injury, *ALT* alanine aminotransferase, *AST* aspartate aminotransferase, *CABG* coronary artery bypass graft, *CPB* cardiopulmonary bypass, *eGFR* estimated glomerular filtration rate, *IABP* intra-aortic balloon pump, *KDIGO* Kidney Disease: Improving Global Outcomes, *LVEF* left ventricular ejection fraction, *LVAD* left ventricular assist device, *MCS* mechanical circulatory support, *NYHA* New York Heart Association, *RRT* renal replacement therapy, *T2DM* type 2 diabetes mellitus, *TAVI* transcatheter aortic valve implantation, *TAVR* transcatheter aortic valve replacement, *UACR* urine albumin creatinine ratio, *ULN* upper limit of normal, *WOCBP* woman of childbearing potential, *WONCBP* woman of non-childbearing potential

### Who will take informed consent? {26a}

The investigator or the investigator’s representative will explain the nature of the study, including the risks and benefits, to the potential participant and answer all questions regarding the study.

Potential participants must be informed that their participation is voluntary. They will be required to sign a statement of informed consent that meets the requirements of ICH guidelines and local regulations such as 21 CFR 50, applicable privacy and data protection requirements, and guidelines of the Institutional Review Boards (IRB), Research Ethics Boards (REB) or Independent Ethics Committees (IEC) or study centre.

The medical record must include a statement that written informed consent was obtained before the participant was enrolled in the study and the date the written consent was obtained. The authorized person obtaining the informed consent must also sign the informed consent form (ICF).

Participants will be reconsented to the most current relevant version of the ICF(s) during their participation in the study. A signed copy of the ICF(s) must be provided to the participant.

### Additional consent provisions for collection and use of participant data and biological specimens {26b}

With participants’ separate consent, samples may be used for further research by sponsor or others such as universities or other companies to contribute to the understanding of kidney injury, changes in renal function, detection and understanding of adverse events of special interest or other diseases, the development of related or new treatments, or research methods.

## Interventions

### Explanation for the choice of comparators {6b}

The double-blind placebo-controlled design allows for an unbiased assessment of efficacy and safety. The use of placebo is justified by the intent to assess absolute efficacy and safety of RMC-035 and is based upon absence of any approved therapeutic drug for reducing the risk of loss of kidney function in cardiac surgery patients.

### Intervention description {11a}

The study intervention RMC-035 or matching placebo, also called investigational medicinal product (IMP), will be delivered by the sponsor as drug concentrate to be diluted by a pharmacist unblinded to the randomised assignment. Administration to study patients according to standard procedures will be performed by trained blinded study site staff. The pharmacist operates separately from the blinded study site team.

Administration of blinded IMP will be done using a syringe pump over a duration of approximately 60 min. Study drug should be administered via a central venous catheter (CVC) whenever possible. One lumen of the CVC should be reserved for IMP administration in order to avoid potential interactions. If a CVC is not available, IMP may also be delivered through a peripheral venous line. Detailed guidelines on the storage, dilution and administration of the study drug are provided in a Pharmacy Manual delivered to each study site.

### Criteria for discontinuing or modifying allocated interventions {11b}

No dose modifications are allowed.

Criteria for permanent discontinuation of IMP include:

- Development of stage ≥ 2 AKI, according to Kidney disease: Improving global outcomes (KDIGO) definition based on local laboratory test results.
- Need for dialysis (continuous and intermittent)
- Need for mechanical circulatory support (MCS) or extracorporeal membrane oxygenation (ECMO).
- Reporting of a grade 3 (moderate intensity) or higher adverse event of infusion site reaction or infusion related reaction.
- Abnormal liver chemistry findings in blood:
  - ◦ Alanine-aminotransferase (ALT) ≥ 3 × upper limit of normal (ULN) and total bilirubin ≥ 2 × ULN without medically plausible alternative explanation
  - ◦ ALT ≥ 3 × ULN if associated with symptoms (new or worsening) believed to be related to hepatitis (such as fatigue, nausea, vomiting, right upper quadrant pain or tenderness or jaundice) or believed to be related to hypersensitivity (such as fever, rash or eosinophilia)
  - ◦ ALT ≥ 3 × ULN and international normalized ratio > 1.5 without medically plausible alternative explanation
  - ◦ ALT ≥ 8 × ULN in any individual sample during the treatment period

A temporary discontinuation of IMP may be considered if, in the opinion of the investigator, a participant's medical condition precludes dosing of study intervention due to a documented serious safety concern. In case of a temporary discontinuation of study intervention, the missed dose will not be replaced or administered at a later time point.

### Strategies to improve adherence to interventions {11c}

All three doses of IMP will be administered under medical supervision in the surgery room, intensive care unit, or on the normal ward by trained study staff; therefore, no additional measures to assure adherence to the intervention protocol are required. Start and stop times of administration as well as volume administered will be documented.

### Relevant concomitant care permitted or prohibited during the trial {11d}

Concomitant therapy is recorded during the study until the end-of-study (EOS) visit. Following the treatment and hospital stay, recording will be limited to relevant medication, eg antihypertensive treatments and drugs known to have an impact on renal hemodynamic, eg sodium glucose co-transporter 2 (SGLT2) inhibitors, angiotensin converting enzyme (ACE) inhibitors, angiotensin II receptor blockers (ARBs), as well as medications related to treat serious adverse events (SAEs) or given in relation to RRT. Detailed instructions on requirement for concomitant therapy collection will be provided in the case report form (CRF) completion guidelines.

Restricted medications include nephrotoxins such as non-steroidal anti-inflammatory drugs and angiotensin converting enzyme (ACE) inhibitors, angiotensin receptor blockers (ARB) and sodium glucose co-transporter 2 (SGLT2) inhibitors, which all are to be discontinued before surgery and not be used during the first 72 h after the first dose of the IMP, unless strictly indicated.

### Provisions for post-trial care {30}

There are no trial-specific provisions for post-trial care in place. The sponsor will ensure that participants are covered by relevant insurances during their participation in the study, in accordance with local laws and regulations. Liability for study induced injury will be covered according to local requirements.

### Outcomes {12}

All endpoints involving eGFR are based on the CKD Epidemiology Collaboration (CKD-EPI) equation of 2021 using SCr assessed in a central laboratory [9]. Calculations of eGFR based on CKD-EPI equations using centrally sampled CysC [12] and a combination of SCr and CysC [9] are used for sensitivity analyses.

#### Primary endpoint

The primary endpoint is the change from baseline in eGFR at Day 90, comparing pooled RMC-035 dose groups to placebo.

In the clinical routine, eGFR is the gold standard for assessment of renal function. It is also an endpoint for irreversible loss of kidney function, a diagnostic marker of CKD, and on the direct path to ESRD [13, 14]. Moreover, a permanent eGFR decline has significant prognostic implications, leading to long-term adverse outcomes on individual patient health, including elevated risk for cardiovascular events, ESRD, and mortality [15–17].

#### Key secondary endpoints

Secondary endpoints of this study include change from baseline in eGFR at Day 90, MAKE_90_, and individual components of MAKE_90_. These endpoints will be assessed using both pooled analyses and dose-level comparisons against placebo.

#### Other secondary endpoints

Other secondary endpoints are based upon pooled, and by dose strength, comparisons of

- Occurrence of MAKE_60_ and each MAKE_60_ component
- Change from baseline in eGFR at Day 7 and Day 60
- Change from baseline in SCr and CysC until Day 7
- Occurrence of AKI based on SCr until Day 4; this includes assessment of AKI stage per KDIGO guideline [10]
- Presence, titre, and cross-reactivity with endogenous A1M, of ADAs at baseline, Day 7, Day 60, and Day 90
- Characteristics of ADA with regards to neutralizing activity and isotype

#### Exploratory endpoints

Changes from baseline in concentrations of markers of complement activation, neutrophil activation, cytokine release and mast cell activation and plasma-concentrations of RMC-035 (area under the curve [AUC], maximum concentration [C_max_] and trough levels [C_trough_]) will be assessed.

#### Safety endpoints

The assessment of safety includes pooled, and by dose strength, comparisons of

- Nature, frequency and severity of treatment emergent adverse events (TEAE); a TEAE is any adverse event (AE) that occurs after start of first administration of IMP and through 72 h (inclusive) after the end of the final administration of IMP
- Nature, frequency and severity of post-treatment AEs (PTAEs); a PTAE is any AE that occurs later than 72 h after the end of the final administration of IMP
- Frequency and severity of TEAEs suggestive of infusion related reactions
- Laboratory test (serum chemistry, haematology and liver function) values and changes from baseline
- Vital signs (blood pressure, pulse rate, respiratory rate, and oxygen saturation), and electrocardiogram (ECG) values and changes from baseline

### Participant timeline {13}

The study consists of 8 visits for each participant, and has three study periods:

- Visit 1: Screening period of 30 days (Day −30 to Day −1)
  - ◦ Day −1, the day before surgery, is a required study timepoint for randomisation and sampling of first baseline SCr
- Visits 2 to 6: Treatment and hospital stay period of approximately 7 days (Day 1 to Day 7)
  - ◦ Day 1 before the patient is prepared for surgery is the required timepoint for sampling of second baseline SCr
  - ◦ Treatment with study drug occurs on Day 1 at 0 and 6 h, and Day 2 at 24 h
  - ◦ Hospital stay continues until discharge at approximately Day 7
- Visits 7 and 8: Follow-up period until Day 90 (from discharge to Day 90)
  - ◦ First follow up visit at 60 days after surgery
  - ◦ Second and last follow up visit at 90 days after surgery

**Fig. 1:**
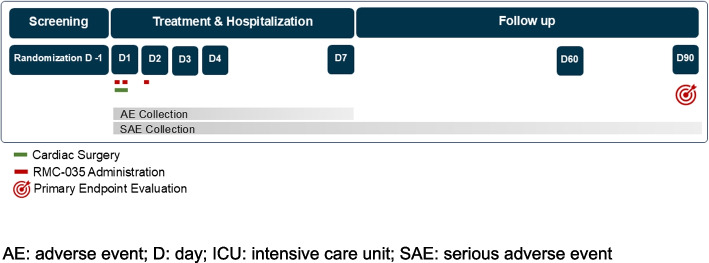
Study flowchart*. *AE: adverse event; D: day; ICU: intensive care unit; SAE: serious adverse event

During the course of the study, visits and assessments will be performed as visualised in the study flow chart [Fig. 1] and as defined in the schedule of assessments for the full study period [Table 3] and for Days 1 and 2 [Table 4].

**Table 3:** Schedule of assessments (Days 1 and 2)

		**Visit 2/Day 1** (Day of surgery)					**Visit 3/Day 2** (EOT)			
**Timepoint**	Pre-0	0	1 h	2 h	Pre-6 h	6 h	Pre-24 h	24 h	25 h	26 h
Pregnancy test	(x)									
Safety labs							x			
SCr *(local lab)*					x		x			
SCr & CysC *(central lab)*	x^1^				x		x			
12-lead ECG							x			
Vital signs	x						x			
IMP administration		x				x		x		
PK *(central lab)*	x		x	x	x		x		x	x
Biomarkers *(central lab)*	x		x		x		x		x	
ADA *(central lab)*	x									
Surgery assessment		During surgery								
RRT recording					As occurs after surgery					
Concomitant Medication recording		As occurs throughout period								
AE/SAE recording		As occurs throughout period								

*ADA* antidrug antibodies, *AE* adverse event, *CysC* cystatin C, *ECG* electrocardiogram, *EOT* end of treatment, *IMP* investigational medicinal product, *SAE* serious adverse event, *PK* pharmacokinetics, *RRT* renal replacement therapy, *SCr* serum creatinine

**Table 4:** Schedule of assessments (full study period)

	**Screening**	**Treatment and hospital stay**					**Follow up**	
**Visit Number** (specification)	**1**	**2** (Day of surgery)	**3** (EOT)	**4**	**5**	**6** (Discharge)	**7**	**8** (EOS)
**Visit Day** (timepoint)	−30 to** −1**	**1**	**2** (24 h)	**3** (48 h)	**4** (72 h)	**7**	**60**	**90**
Informed consent	x							
Eligibility check	x							
Medical & surgical history, incl. substance use	x							
Demographics	x							
Physical examination	x			x				
Weight and (screening only) height	x						x	x
Pregnancy test (WOCBP only)	x	(x)						
Safety lab tests *(local lab)*	x		x	x	(x)	(x)		
SCr test *(local lab)*	x	x	x	x	x	x		
SCr & CysC test *(central lab)*	x	x	x	x	x	x	x,x	x,x
Randomisation	x							
12-lead ECG	x		x	x				
Vital signs	x	x	x	x				
IMP administration		x	x					
PK test *(central lab)*		x	x	**x**				
Biomarkers test *(central lab)*		x	x	x				
ADA test *(central lab)*		x				x	x	x
Surgery assessments		x						
RRT		As occurs after surgery to EOS						
Concomitant medication		As occurs from Day 1 to EOS						
AE recording		As occurs from first dose to discharge						
SAE recording		As occurs from first dose to EOS						

*ADA* antidrug antibodies, *AE* adverse event, *CysC* cystatin C, *ECG* electrocardiogram, *EOS* end of study, *EOT* end of treatment, *IMP* investigational medicinal product, *SAE* serious adverse event, *PK* pharmacokinetics, *RRT* renal replacement therapy, *SCr* serum creatinine, *SoA* schedule of assessment, *WOCBP* woman of childbearing potential

### Sample size {14}

A total of 161 participants will be randomised in a 2:2:3 ratio across three study arms: 46 for RMC-035 low dose, 46 for RMC-035 high dose, and 69 for placebo.

This design aims to achieve at least 80% power to detect a significant effect of pooled dose levels compared to placebo at a 10% two-sided significance level, assuming a treatment effect of 5 mL/min/1.73m^2^ and a standard deviation of 12 mL/min/1.73m^2^. The expected effect size is conservatively based on observations from the AKITA study in the subgroup with eGFR < 60 mL/min/1.73m^2^, where a lower starting dose was administered [8]. In this subgroup, descriptive analysis indicated a difference in eGFR of 6.5 mL/min/1.73m^2^, while a mixed model of repeated measures (MMRM) showed 7.9 mL/min/1.73m^2^. The standard deviation was derived from the AKITA study, which reported approximately 13 mL/min/1.73m^2^. To minimise variability, double assessment of centrally sampled serum creatinine at baseline, days 60 and 90 will be used for eGFR calculation, with an expected standard deviation of 12 mL/min/1.73m^2^ in the current study. A 10% drop-out rate is anticipated for the 90-day assessment, along with a 5% information loss.

### Recruitment {15}

Participants will be recruited from the pool of patients being scheduled for relevant open-heart procedures at the selected study sites. Study sites were mainly selected based upon enrolment experience in the previous phase 2 AKITA study.

## Assignment of interventions: allocation

### Sequence generation {16a}

Participants are randomised block wise in a 2:2:3 ratio to RMC-035 high dose, RMC low dose, and placebo, respectively. Stratification for eGFR (≥ 60 and < 60 mL/min/1.73m^2^) will be implemented, and at least 30% of participants will be included in the < 60 mL/min/1.73m^2^ stratum.

### Concealment mechanism {16b}

Stratified randomisation to IMP is done centrally using an interactive response technology (IRT) based upon patient information entered by the study site staff: age, gender and SCr. Blinded study site staff does not become aware of the intervention assignment.

### Implementation {16c}

The allocation sequence is generated by a vendor commissioned by the study sponsor. Enrolment and assignment of study participants to study intervention is done by trained study site staff; relevant user directions for the use of the IRT system are provided to each site.

## Assignment of interventions: blinding

### Who will be blinded {17a}

Study participants, investigators and clinical site staff involved in enrolment, treatment and data collection, and sponsor representatives, are blinded to study intervention. The unblinded investigational pharmacist or an appropriately qualified person responsible for the preparation of study intervention for each participant is informed via automated message from the IRT about the intervention allocation. Unblinding to the randomisation assignment is restricted to this staff only. Study documentation at the pharmacy is kept separate from other study documentation.

### Procedure for unblinding if needed {17b}

The IRT will be programmed with blind-breaking instructions. In case of an emergency, the investigator has the sole responsibility for determining if unblinding of a participant’s intervention assignment is warranted. Participant safety must always be the first consideration in making such a determination. If the investigator decides that unblinding is warranted, the investigator may, at the investigator’s discretion, contact the sponsor to discuss the situation prior to unblinding a participant’s intervention assignment unless this could delay emergency treatment for the participant.

Sponsor or sponsor delegated safety staff may unblind the intervention assignment for any participant with an SAE. If the SAE requires that an expedited regulatory report be sent to one or more regulatory agencies, a copy of the report, identifying the participant’s intervention assignment, may be sent to IRBs, REBs or IECs in accordance with local regulations.

An independent Data Safety Monitoring Committee (DSMC) will access masked or unblinded data during the study; see section Oversight and monitoring. This will have no impact on the blinding for participants, investigators and clinical site staff, or sponsor representatives.

## Data collection and management

### Plans for assessment and collection of outcomes {18a}

All data required will be recorded by trained study site staff into an electronic CRF (eCRF) accessible via an electronic data entry portal set up by a vendor delegated by the study sponsor. Access to the data entry function of the portal is limited to trained study staff who are granted individual password-protected access rights. Guidance on data entry is provided in study-specific eCRF completion guidelines.

### Plans to promote participant retention and complete follow-up {18b}

Following surgery and hospital discharge patients will be followed up at Days 60 and 90 after the first dose of study intervention. Trained study staff will keep contact with patients and remind them of attending the follow-up visits at the study site. To ease patient retention and completeness of data collection during the follow-up period these visits may be performed by qualified and trained study staff at the investigational site, at the participant's home, or other suitable location, where appropriate.

If study intervention is discontinued, the participant should still remain in the study to be evaluated for all assessments during treatment and hospital stay period as well as follow-up through to Day 90.

A participant may withdraw from the study at any time at the participant’s own request for any reason (or without providing any reason) or at the discretion of the investigator for safety reasons. At the time of discontinuing from the study, if possible and appropriate, the participant will be asked if they want to withdraw from treatment but are willing to accept continued follow up assessments. At a minimum, if appropriate, the participant should be asked if a phone call at Day 90 is acceptable to follow up on any need for RRT and possible SAEs. Where permitted by local regulations, publicly available data (eg, death records) can be included after withdrawal of consent.

### Data management {19}

Details for data entry into the eCRF, coding, security and storage of data and a description of processes related to assure data quality can be found in a separate data management plan set up by the sponsor delegate and approved by the sponsor. This plan describes the receipt and management of study data from start-up to close-out of the study. This plan also outlines the responsibilities of the applicable staff delegated by the sponsor.

### Confidentiality {27}

Participants will be assigned a unique identifier by the sponsor. Any participant records or datasets that are transferred to the sponsor will contain the identifier only; participant names or any information which would make the participant identifiable will not be transferred. The participants are informed that their personal study-related data will be used by the sponsor in accordance with local data protection law. The level of disclosure must also be explained to the participant who will be required to give consent for their data to be used as described in the ICF. Collection of personal data, also including race and ethnicity, is for scientific research purposes and in accordance with Article 89 of the General Data Protection Regulation (GDPR) and §27 of the Data Protection Adaptation and Implementation Act EU of 30 June 2017. The participant must be informed that their medical records may be examined by clinical quality assurance auditors or other authorized personnel appointed by the sponsor, by appropriate IRB/REB/IEC members, and by inspectors from regulatory authorities.

The contract between sponsor and study sites specifies responsibilities of the parties related to data protection, including handling of data security breaches and respective communication and cooperation of the parties.

Information technology systems used to collect, process, and store study-related data are secured by technical and organizational security measures designed to protect such data against accidental or unlawful loss, alteration, or unauthorized disclosure or access.

### Plans for collection, laboratory evaluation and storage of biological specimens for genetic or molecular analysis in this trial/future use {33}

There are no plans to collect, store and evaluate biological specimens for genetic or molecular analysis.

## Statistical methods

### Statistical methods for primary and secondary outcomes {20a}

Full details of the statistical methodology are described in a statistical analysis plan separate from the study protocol.

#### Primary endpoint (main analytical approach)

The eGFR change from baseline to Day 90 will be estimated using a MMRM. The change from baseline values for post baseline measurements will be modelled in the response vector and group (pooled RMC-035 and placebo), time and group*time as fixed factors. Participant will be included as a random factor in the model. The estimated difference between the pooled RMC-035 group and placebo at each time point will be calculated, along with the respective 90% confidence interval (CI) and *p*-value, where the estimated difference at the 90-day visit is defined as the primary endpoint. The estimated mean change from baseline per arm and respective 90% CI and *p*-value will also be calculated. An unstructured covariance matrix will be used to model dependence between time-points.

#### Key secondary endpoints (man analytical approach)

Occurrence of MAKE_90_ and its components will be analysed using a Cochran-Mantel–Haenszel model to estimate of the common relative risk (pooled RMC-035 versus placebo) across the stratification group formed by eGFR at Day −1 (≥ 60 and < 60 mL/min/1.73m^2^). The estimate of the common relative risk, 90% CI utilizing Greenland and Robins, and *p*-value will be reported. The proportion of participants with MAKE_90_ and each MAKE_90_ component, and its 90% CI will be calculated.

Change from baseline in eGFR at Day 90 (by each RMC-035 dose level) will be analysed using the same model as the primary endpoint, but where treatment will include both dose levels of RMC-035 and difference versus placebo will be estimated for each dose level.

Occurrence of MAKE_90_ and its components by each RMC-035 dose level will be analysed using the same model as for the pooled treatment group analyses, but where treatment will include both dose levels of RMC-035 and relative risk versus placebo will be estimated for each dose level.

#### Other secondary endpoints and exploratory endpoints

Analytical approaches for other secondary and for exploratory endpoints are described in [*Supplement 1*].

#### Safety endpoints

- All safety analyses will be summarized by treatment arm (Placebo, pooled RMC-035 [30 mg and 60 mg], and separately for 30 mg and 60 mg)
- Number and proportions of patients with AEs, TEAEs and PTAEs will be summarized including serious, fatal and severity grade ≥ 3 events.
- Adverse events of special interest (AESI):
  - ◦ Summary by treatment group of number and proportions of patients with and number and proportions of TEAEs suggestive of
    - ▪ Infusion related reactions (overall and by preferred term)
    - ▪ Infusion site reactions grade 3 or higher
  - ◦ Analysis of onset of AESI relative to prior infusion
- Descriptive statistics will be provided for laboratory tests (haematology and biochemistry) and vital signs (pulse rate, respiratory rate and blood pressure) by visit and for the changes from baseline to each visit.
- Continuous ECG interval measurements will be summarized by parameter, treatment group and analysis visit. Actual values and changes from baseline will be tabulated separately by each visit. This tabulation will include the maximum and the minimum value post-baseline.
- Shift tables will be produced for potentially clinically significant ECG (worst values):
  - ◦ QTcF (ms): > 450, > 480, > 500
  - ◦ Change from baseline in QTcF (ms): > 30, ≥ 60

### Interim analyses {21b}

There will be no formal interim analysis of efficacy endpoints in this study. Interim safety data will be reviewed in an unblinded manner by a DSMC; see section Monitoring and oversight.

### Methods for additional analyses (e.g. subgroup analyses) {20b}

Subgroup analyses of the primary endpoint and secondary endpoints will be performed to assess consistency of the investigational intervention effect across the following subgroups:

- eGFR at Screening < 60 mL/min/1.73m^2^ versus ≥ 60 mL/min/1.73 m.^2^
- Diabetes mellitus at Screening: Yes and No
- Region: North America and Europe
- Gender: Male and Female

If the number of participants is too small (less than 10%) within a subgroup, then the subgroup categories may be redefined prior to unblinding the study. The analyses will be conducted using a test for heterogeneity and results will be presented on forest plots presenting the estimated study arm difference and 90% confidence intervals.

#### Primary endpoint (sensitivity analyses)

The same model as the primary analysis will be repeated and adjusted for use of SGLT2 inhibitors, ACE inhibitors, or ARBs, where the covariate will be 0 for all time points until first exposure, and 1 for all time points thereafter. For additional sensitivity analyses, the same model as the primary analysis will be used where eGFR is based on SCr combined with CysC and based on CysC alone.

#### Key secondary endpoints (sensitivity analyses)

Absolute eGFR change from baseline at Day 90, and MAKE_90_ including its components, with eGFR based on combined SCr and CysC (CKD-EPI formula 2021) and based on CysC (CKD-EPI formula 2012) for pooled RMC-035 and for each RMC-035 treatment arm versus placebo.

Occurrence of modified MAKE_60_/MAKE_90_ (i.e., RRT, ≥ 25% decline in eGFR) will be analysed as described in Key secondary endpoints.

### Methods in analysis to handle protocol non-adherence and any statistical methods to handle missing data {20c}

Missing data for primary and secondary endpoints may be the result of intercurrent events. As a general approach a treatment policy strategy will be pursued for eGFR change from baseline (primary endpoint, key secondary endpoint) using the MMRM model in all participants randomised and treated with at least one dose.

For the key secondary endpoint involving occurrence of MAKE a treatment policy strategy will be pursued, regardless of missing eGFR data.

For other efficacy endpoints values for missing data will not be imputed.

Conventions will be made for missing data for start/stop times/dates of AEs or concomitant medication.

Protocol deviations will be defined in a protocol deviation plan separate from the study protocol. A final review and reconciliation of protocol deviations will be performed prior to database lock. Counts and percentages of participants with major protocol deviations by deviation category will be summarized by treatment arm based on all randomised participants.

### Plans to give access to the full protocol, participant level-data and statistical code {31c}

Details of the study protocol including statistical considerations are available at clinicaltrials.gov (NCT06475274). The full protocol and individual participant data will not be shared.

## Oversight and monitoring

### Composition of the coordinating centre and trial steering committee {5d}

There is no specific steering committee for this phase 2b dose-finding trial.

### Composition of the data monitoring committee, its role and reporting structure {21a}

An independent DSMC, comprising of two experts in nephrology and anaesthesiology, and an independent statistician, will oversee participant safety once approximately 33% of the participants have been treated and completed Day 7 and again when approximately 67% of participants have reached this timepoint. The DSMC may also conduct ad hoc reviews at any time. They will have access to both masked and unblinded data for their assessments and will provide recommendations for the study's conduct following their meetings, in accordance with a pre-specified DSMC charter on file with the sponsor.

### Adverse event reporting and harms {22}

An AE is any untoward medical occurrence in a clinical study participant, temporally associated with the use of study intervention, whether or not considered related to the study intervention. This includes any abnormal laboratory test results or other safety assessments, including those that worsen from baseline, considered clinically significant in the medical and scientific judgment of the investigator or an exacerbation of a chronic or intermittent pre-existing condition.

The investigator and any qualified designees are responsible for detecting, documenting, and recording events that meet the definition of an AE or SAE and remain responsible for following up all AEs (clinical, laboratory values, or other) until the return to normal, or until the participant’s condition has stabilized. In the event of an SAE, the Investigator will follow-up with the outcome until the clinical recovery is complete and laboratory results have returned to normal or until progression has been stabilized. This includes events reported by the participant (or, when appropriate, by a caregiver).

All non-serious AEs will be collected from the start of study intervention until Day 7. All SAEs will be collected from the start of study intervention until Day 90.

Care will be taken not to introduce bias when detecting AEs. Open-ended and nonleading verbal questioning of the participant or caregiver is the preferred method to inquire about AE occurrences.

### Frequency and plans for auditing trial conduct {23}

Independent audits will be carried out by the sponsor or a delegate of the sponsor. The conduct of routine and ad-hoc audits and selection criteria for sites to be audited are specified in a separate audit plan.

### Plans for communicating important protocol amendments to relevant parties (e.g. trial participants, ethical committees) {25}

The protocol, protocol amendments, ICF, Investigator’s Brochure, and other relevant documents (eg, advertisements) must be submitted to an IRB/REB/IEC by the investigator and reviewed and approved by the IRB/REB/IEC before the study is initiated at any particular site. Any amendments to the protocol will require IRB/REB/IEC approval before implementation of changes made to the study design, except for changes necessary to eliminate an immediate hazard to study participants. Protocols and any substantial amendments to the protocol will also require health authority approval prior to initiation except for changes necessary to eliminate an immediate hazard to study participants.

## Dissemination plans {31a}

Results from the study will be presented in a clinical study report as per International Council for Harmonisation of Technical Requirements for Pharmaceuticals for Human Use (ICH) E3 guideline and will be disclosed next to periodic safety reports, and clinical study summary reports as required by applicable regulations. Study information and tabular study results will be posted on the US National Institutes of Health’s website www.clinicaltrials.gov and the EU Clinical Trials register https://euclinicaltrials.eu/. The study results will also be published in peer-reviewed journals.

## Discussion

This phase 2b study, POINTER, aims to confirm the efficacy signal observed in the previous phase 2a (proof-of-concept) AKITA study, which assessed the efficacy of RMC-035 – a modified human therapeutic A1M protein – in preserving renal function in cardiac surgery patients at high risk for kidney injury.

Based on the AKITA study results, the dosing regimen of RMC-035 has been optimized in POINTER by streamlining infusion time, reducing the number of infusions, and transitioning from a weight-based to a fixed dose, irrespective of preoperative body weight and renal function. The benefit-risk profile of each RMC-035 dose level in POINTER will be evaluated to determine the optimal dose for a pivotal phase 3 study.

Notably, the primary endpoint, eGFR change from baseline, and key secondary endpoints such as MAKE, are assessed at Day 90. This approach enables the evaluation of renal function in a stable phase after surgery, minimizing the influence of peri- and postoperative confounders. Furthermore, this approach is favored by regulatory authorities and represents a paradigm shift in “AKI trials”, which traditionally assess interventions based on short-term endpoints, such as AKI defined by KDIGO [17] or the recommendation to use AKI as primary endpoint in prevention trials [18], supported by observational trial data linking AKI occurrence and/or postoperative SCr increase with adverse long-term renal outcomes [3, 10, 18]. However, emerging data has increasingly highlighted the limitations of short-term endpoints:

- The degree of renal impairment, presence or absence of cellular injury, and potential for irreversible loss of kidney function cannot be sufficiently discriminated due to the absence of appropriate diagnostic tools, such as kidney biopsies or robust biomarkers [2].
- Tissue injury predisposing to permanent renal function loss may be obscured by postoperative improvements in cardiac output and renal blood flow, as well as the activation of functional renal reserve. This can mask nephron loss through hyperfiltration within the remaining nephrons [3].
- Minor and transient increases in SCr, reaching AKI stage 1 or remaining below this threshold – most commonly observed after surgery and often attributed to hemodynamic changes – are likely unmodifiable.
- Event rates for clinically meaningful short-term renal outcomes, such as RRT and/or severe AKI, even in high-risk patient groups, are typically below 10%. A robust assessment of these outcomes before a large pivotal phase 3 study is therefore infeasible.

These points are also reinforced by recent data from both interventional and observational AKI trials, demonstrating that many cardiac surgery patients who do not meet formal AKI criteria still experience irreversible and clinically significant eGFR loss, and vice versa [19, 20].

Integrating these perspectives, it is deemed more meaningful to assess kidney-protective treatments, whether preventive or interventional, based on longer-term renal outcomes. In cardiac surgery, up to 30% of patients experience a clinically significant and sustained decline in eGFR during the stable phase after surgery [21]. From a regulatory standpoint, MAKE_90_ – a composite endpoint including death, initiation of renal replacement therapy, or a ≥ 25% decline in eGFR – is considered a meaningful, patient-centered measure for evaluating kidney benefits in a pivotal phase 3 study in the cardiac surgery setting. However, the dichotomous nature of MAKE_90_, with a fixed eGFR threshold of ≥ 25% loss, is not practical for establishing clinical proof-of-concept or determining the target dose in phase 3 due to the large sample size required for a binary endpoint with a low event rate.

The POINTER trial addresses these limitations by using eGFR change from baseline to Day 90 as the primary endpoint. eGFR is the gold standard for assessing kidney function in clinical practice, and its change over time is considered a viable intermediate endpoint, likely reflecting a new steady state after surgery and indicating permanent kidney function loss. In CKD trials, eGFR slope (annualized eGFR decline) has been shown to be a reliable predictor of and directly linked to ESRD. Although CKD and AKI trials differ (i.e., CKD trials assess eGFR decline over at least two years, while AKI trials typically measure a “one-time” injury and eGFR change within 90 days) it is reasonable to consider eGFR as an appropriate renal endpoint in the current dose-finding study. This approach is considered superior to any acute SCr-based endpoints.

In the predecessor AKITA study, the use of an acute primary endpoint was necessary due to the lack of clinical and preclinical data supporting meaningful efficacy assumptions for RMC-035 at 90 days post-surgery. The AKITA results indicated a treatment benefit of RMC-035 compared to placebo greater than 6 mL/min/1.73m^2^ when avoiding overexposure [8]. Based on these findings, a data-driven assumption on efficacy was made for POINTER, using a conservative efficacy estimate of 5 mL/min/1.73m^2^.

The strengths of the study are based upon its randomized, placebo-controlled, double-blind study design in an international multicentre setting reflecting heterogeneity in pre- and postoperative management of cardiac surgery patients. To assure completeness of data and to minimise missing data all patients will be followed up by Day 60 and Day 90. Study participants will either present directly at the site for follow-up or be visited at home or in their rehabilitation centre by a trained remote study nurse. Blood samples for analyses of SCr as basis for eGFR estimation will be assessed in a central lab and are complemented by centrally assessed CysC for the purposes of sensitivity analyses considering the impact of factors like potential variability in body and muscle mass on SCr.

The POINTER study design also has limitations. A predefined two-sided alpha of 0.1 is applied, which provides less stringent alpha control than required in a pivotal setting. However, this level is considered appropriate given the study objectives and in light of the AKITA results, which were based on a similar design as POINTER. The study is not powered for secondary endpoints like MAKE_90_, and no multiplicity adjustment is applied. Also, despite the clinical arguments in support of eGFR change as primary endpoint, this intermediate endpoint is not validated as prognostic indicator for MAKE or other long-term kidney outcomes.

In conclusion, this multicentre multinational phase 2b study, named POINTER, will assess change in eGFR within 90 days of first dose and thereby provide additional evidence of the kidney-protective potential of RMC-035 in patients undergoing cardiac surgery and at high risk for kidney injury. Study outcomes will inform the preferred dose, dosing regimen, and benefit-risk profile related to cardiac surgery for a future pivotal phase 3 study.

## Trial status

The current protocol version number is 2.1 dated July 2, 2024. The trial is open for enrolment and ongoing. The first participant was enrolled August 26, 2024. The last patient was enrolled on June 03, 2025.

## Supplementary Information

Supplementary Material 1.

## Data Availability

The sponsor and its delegates will have access to the full study database. At the end of the study each study site will receive a copy of the patient data entered by the respective site.

## Abbreviations

A1M: Alpha-1-microglobulin
ACE: Angiotensin converting enzyme
ADA: Antidrug antibody
AE: Adverse event
AESI: Adverse event of special interest
AKI: Acute kidney injury
ALT: Alanine aminotransferase
ARB: Angiotensin receptor blocker
AUC: Area under the curve
CABG: Coronary artery bypass graft
CI: Confidence interval
CKD: Chronic kidney disease
CKD-EPI: CKD Epidemiology Collaboration
CKD-EPI: CKD Epidemiology Collaboration
C_max_: Maximum concentration
CPB: Cardiopulmonary bypass
CRF: Case report form
C_trough_: Trough level
CVC: Central venous catheter
CysC: Cystatin C
DSMC: Data Safety Monitoring Committee
ECG: Electrocardiogram
ECMO: Extracorporeal membrane oxygenation
eCRF: Electronic CRF
eGFR: Estimated glomerular filtration rate
ESRD: End-stage renal disease
GDPR: General Data Protection Regulation
ICF: Informed consent form
ICH: International Council for Harmonisation of Technical Requirements for Pharmaceuticals for Human Use
IEC: Independent Ethics Committee
IMP: Investigational medicinal product
IRB: Institutional Review Board
IRT: Interactive response technology
KDIGO: Kidney disease: Improving global outcomes
MAKE: Major adverse kidney event
MAKE_60_: MAKE at Day 60
MAKE_90_: MAKE at Day 90
MCS: Mechanical circulatory support
MMRM: Mixed model of repeated measures
PTAE: Post-treatment adverse event
REB: Research Ethics Board
ROS: Reactive oxygen species
RRT: Renal replacement therapy
SAE: Serious adverse event
SCr: Serum creatinine
SGLT2: Sodium glucose co-transporter 2
TEAE: Treatment-emergent adverse event
ULN: Upper limit of normal
