# Supplementary material for: POINTER: study protocol for a phase 2b, randomised, placebo-controlled, double-blind, parallel group dose-finding clinical study to evaluate the efficacy of RMC-035 on renal function and safety, in participants at high risk for kidney injury, following open-chest cardiac surgery
Source: Trials. 2025 Oct 28;26:449. doi: 10.1186/s13063-025-09124-x (PMC12570843; doi:10.1186/s13063-025-09124-x)
Supplement: Supplementary file 1 — Supplementary Material 1. [file 13063_2025_9124_MOESM1_ESM.docx]

**Supplement 1 Statistical analyses of other secondary endpoints and exploratory endpoints**

*Other secondary endpoints (main analytical approach)*

The following endpoints will be analysed using the same method as the corresponding primary or key secondary endpoint:

- Occurrence of MAKE_60_ and its components (pooled RMC-035 dose levels).
- Occurrence of MAKE_60_ and its components (by each RMC-035 dose level).
- Change from baseline in eGFR at Day 7 and Day 60 (pooled RMC-035 dose levels) will be analysed using the same method as the primary endpoint.
- Change from baseline in eGFR at Day 7 and Day 60 (by each RMC-035 dose level).
- Occurrence of AKI by Day 4 (pooled RMC-035 dose levels) based on SCr.
- Occurrence of AKI by Day 4 (by each RMC-035 dose level) based on SCr.

The following endpoints will be analysed using descriptive statistics:

- Occurrence of AKI by Day 4 by stages 1, 2 and 3.
- SCr and CysC values and their absolute changes from baseline until Day 7.
- Presence, titre, and cross-reactivity with endogenous A1M, of ADAs at Baseline, Day 7, Day 60 and Day 90.
- Characteristics of ADA with regards to neutralising activity and isotype.

*Other secondary endpoints (sensitivity analyses)*

Occurrence of AKI by Day 4 (72h) (pooled RMC-035 dose levels and by each dose level) based on CysC will be analysed using the same method as the key secondary endpoint.

*Exploratory endpoints*

The following endpoints will be analysed using descriptive statistics:

- Concentrations and changes from baseline of markers of complement activation, neutrophil activation, cytokine release and mast cell activation for pooled RMC-035, and for each RMC-035 dose level, versus placebo.
- Plasma-concentrations of RMC-035, AUC, C_max_ and C_trough_ for each RMC-035 dose level.
